# Therapeutic MK2 inhibition blocks pathological vascular smooth muscle cell phenotype switch

**DOI:** 10.1172/jci.insight.142339

**Published:** 2021-10-08

**Authors:** J. William Tierney, Brian C. Evans, Joyce Cheung-Flynn, Bo Wang, Juan M. Colazo, Monica E. Polcz, Rebecca S. Cook, Colleen M. Brophy, Craig L. Duvall

**Affiliations:** 1 Department of Biomedical Engineering, Vanderbilt University, Nashville, Tennessee, USA.; 2 Division of Vascular Surgery, Department of General Surgery, Vanderbilt University Medical Center, Nashville, Tennessee, USA.; 3 Medical Scientist Training Program, Vanderbilt University School of Medicine, Nashville, Tennessee, USA.; 4 Department of General Surgery and; 5 Vanderbilt-Ingram Cancer Center, Vanderbilt University Medical Center, Nashville, Tennessee, USA.

**Keywords:** Vascular Biology, Cardiovascular disease, Drug therapy

## Abstract

Vascular procedures, such as stenting, angioplasty, and bypass grafting, often fail due to intimal hyperplasia (IH), wherein contractile vascular smooth muscle cells (VSMCs) dedifferentiate to synthetic VSMCs, which are highly proliferative, migratory, and fibrotic. Previous studies suggest MAPK-activated protein kinase 2 (MK2) inhibition may limit VSMC proliferation and IH, although the molecular mechanism underlying the observation remains unclear. We demonstrated here that MK2 inhibition blocked the molecular program of contractile to synthetic dedifferentiation and mitigated IH development. Molecular markers of the VSMC contractile phenotype were sustained over time in culture in rat primary VSMCs treated with potent, long-lasting MK2 inhibitory peptide nanopolyplexes (MK2i-NPs), a result supported in human saphenous vein specimens cultured ex vivo. RNA-Seq of MK2i-NP–treated primary human VSMCs revealed programmatic switching toward a contractile VSMC gene expression profile, increasing expression of antiinflammatory and contractile-associated genes while lowering expression of proinflammatory, promigratory, and synthetic phenotype–associated genes. Finally, these results were confirmed using an in vivo rabbit vein graft model where brief, intraoperative treatment with MK2i-NPs decreased IH and synthetic phenotype markers while preserving contractile proteins. These results support further development of MK2i-NPs as a therapy for blocking VSMC phenotype switch and IH associated with cardiovascular procedures.

## Introduction

A common complication associated with angioplasty and vascular grafting procedures is intimal hyperplasia (IH), characterized by rampant vascular smooth muscle cell (VSMC) proliferation and abundant extracellular matrix (ECM) deposition, resulting in excessive neointimal formation, stenosis, and conduit failure (1–3). Current strategies to mitigate IH utilize antiproliferative drugs, such as the chemotherapeutic agent paclitaxel (4) or the mTOR inhibitor sirolimus (5), which are integrated into drug-eluting stents and angioplasty balloons for vascular wall delivery (6–8). Even with these interventions in place, nearly half of all grafts and angioplasties require further intervention because of IH. Increasing evidence suggests that antiproliferative treatments alone may impede graft reendothelialization, contributing to late-stage thrombosis (9). Agents specifically targeting VSMC proliferation, such as the E2F decoy edifoligide, do not provide an overall clinical benefit in preventing thrombosis and vessel failure or supporting vessel patency (10–12). An increased understanding of the molecular mechanisms regulating VSMC phenotypic switching is needed.

In healthy, mature vessels, contractile VSMCs of the medial layer undergo negligible proliferation and express abundant contractile proteins (e.g., α–smooth muscle actin, α-SMA) to regulate vasoconstriction and vasodilation. Mechanical stress and molecular pressures occurring in wounded or manipulated vascular tissues, including inflammation and growth factor signaling, initiate a reversible VSMC transition toward a synthetic phenotype. The increased proliferation and ECM deposition exhibited by synthetic VSMCs support vascular wound-healing processes for neointima repair and vessel remodeling (13–16). Interestingly, the contractile-to-synthetic phenotype switch may become self-sustaining through molecular pathways that are only recently being discovered, such as expression of the fibronectin isoform Fn-EDA and innate immune receptor signaling via TLR4 (17). Sustained persistence of the synthetic VSMC phenotype leads to IH and ultimate failure of cardiovascular interventions.

Although the molecular events perpetuating the synthetic VSMC phenotype remain unclear, it is possible that, once identified, blockade of these signaling pathways will reduce IH in the setting of angioplasty or vascular grafts. This notion is supported by studies showing that delivery of exogenous miRNA-145, responsible for downregulation of proliferation-inducing transcription factors (e.g., *KLF4* and *ELK1*) (18), successfully inhibits proliferation and enhances contractile differentiation in VSMC models and reduces neointimal thickness in saphenous vein cultures (19, 20). Notably, miRNA-145 is potently downregulated in proliferating VSMCs (19, 21) and is itself regulated by the transcription factor myocardin/serum response factor, which is prevalent in contractile VSMCs.

An intense interest in the druggable signaling pathways that drive IH has identified a central role for the stress-activated kinase p38 MAPK, although its role in VSMC phenotype switching, per se, remains unresolved. Interestingly, the serine-threonine kinase MAPK-activated protein kinase 2 (MK2) is directly activated by p38 MAPK in VSMCs upon mechanical or molecular stress (22), allowing MK2 to phosphorylate several factors that promote VSMC proliferation (23–29). MK2 inhibition blocks VSMC proliferation and reduces neointimal formation. Here, we demonstrated that delivery of an MK2 inhibitory peptide (MK2i) blocks the gene expression changes that determine contractile-to-synthetic VSMC dedifferentiation, thus preventing VSMC proliferation, inflammatory signaling, and ECM production. By blocking synthetic VSMC functions, MK2i therapy helps prevent IH and preserve vessel patency.

## Results

### Modulation of primary SMC phenotype marker expression in vitro.

**Figure 1:**
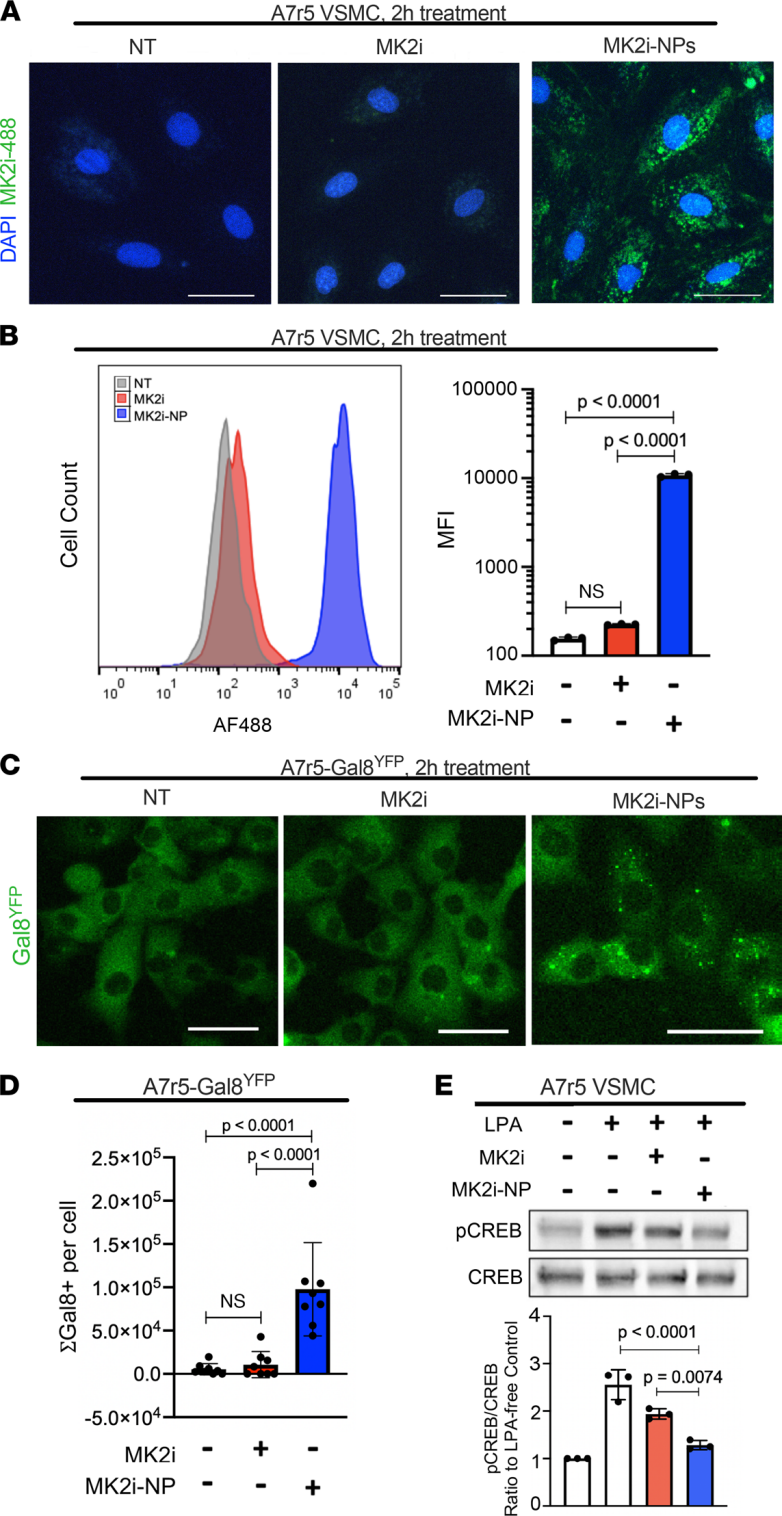
MK2i-NPs increase cell uptake and endosomal escape of MK2i in A7r5 cells while maintaining bioactivity. A7r5 cells were incubated for 2 hours with vehicle control (no treatment, NT) or 50 μM MK2i or MK2i-NPs^50:5Lyo^ made with MK2i conjugated with Alexa Fluor 488. (**A**) After 2 hours, A7r5s were imaged under fluorescence microscopy to confirm uptake of MK2i-488. (**B**) Flow cytometry was used to quantify cell uptake of MK2i, and MFI for 3 replicates was quantified. (**C**) A7r5 cells engineered to express YFP-Gal8 were treated with 50 μM MK2i-488 or MK2i-NPs^50:5Lyo^ and imaged to visualize endosomal disruption 3 hours after treatment. (**D**) The total Gal8-positive pixels were normalized to cell number for 8 replicates. (**E**) A7r5 cells were treated with 50 μM MK2i or MK2i-NPs (50 μM MK2i and 2.5 μM PPAA) and cultured for 24 hours after treatment prior to LPA stimulation and protein harvesting for Western blot analysis of CREB phosphorylation. *n* = 3. All statistics were determined using 1-way ANOVA and Tukey’s multiple-comparison test. Scale bars: 50 μm.

To assess the role of MK2 in VSMC transdifferentiation, we employed a previously characterized MK2i, which binds the MK2 catalytic site to block its kinase activity (30). The positively charged MK2i was electrostatically complexed with poly(propyl acrylic acid) (PPAA), a negatively charged polymer that potentiates intracellular bioavailability and activity of cationic cargoes (31, 32), in a 50 μM:5 μM MK2i/PPAA ratio, thus generating MK2i-nanopolyplexes (MK2i-NPs). Importantly, previous reports show that PPAA has no impact on cell viability in vitro (31) and in human saphenous vein (HSV) cultures (33). This formulation was made at 10× and lyophilized in excipient lactosucrose (300 mM) to stabilize MK2i-NPs and enhance their activity in tissues (34). This formulation, referred to here as MK2i-NP^50:5Lyo^, was delivered to embryonic rat A7r5 VSMCs for 2 hours. Alexa Fluor 488–labeled MK2i-NP^50:5Lyo^ was taken up by A7r5 cells to a greater extent than Alexa Fluor 488–labeled free MK2i delivered at similar molar dose (50 μM), as shown by fluorescence microscopy (Figure 1A) and flow cytometry, revealing significantly more Alexa Fluor 488–positive cells and 153-fold higher MFI per cell (Figure 1B) in samples treated with MK2i-NP^50:5Lyo^ over untreated or free MK2i-treated cells. Additionally, uptake of MK2i was improved by a nanopolyplex formulation complexed in a 50:2.5 molar ratio (Supplemental Figure 1; supplemental material available online with this article; https://doi.org/10.1172/jci.insight.142339DS1), a preparation also used herein.

Once taken up, MK2i-NPs would need to access their target, MK2, within the cytoplasm, thus requiring endosomal escape (35). This was measured in A7r5 cells expressing galectin 8 (Gal8), a cytoplasmic protein redistributing and clustering onto the interior surface of disrupted endosomes, tagged with yellow fluorescent protein (Gal8^YFP^) (Figure 1C). Endosomal Gal8^YFP^ puncta were 9.1 times more prevalent in MK2i-NP^50:5Lyo^-treated A7r5 cells over untreated or MK2i-treated cells (Figure 1D), confirming potent endosomal disruption by MK2i-NP^50:5Lyo^. Previous studies have demonstrated the positive correlation between endosomal Gal8^YFP^ puncta and cytoplasmic bioavailability of NP cargo (36). Inhibition of MK2 activity by MK2i-NP was determined by measuring phosphorylation of the direct MK2 target cAMP response element–binding protein (CREB) (25), a transcription factor involved in VSMC proliferation (27, 28). As expected, CREB phosphorylation was potently induced by lysophosphatidic acid (LPA) in A7r5 cells (Figure 1E). However, treatment with MK2i-NP^50:5Lyo^ reduced LPA-induced CREB phosphorylation nearly 75% (Figure 1E), significantly better than what was achieved using free MK2i. Together, these studies confirmed intracellular MK2 and bioactivity of the MK2i-NPs.

### MK2i-NP treatment blocks PDGF-induced proliferation in VSMCs.

**Figure 2:**
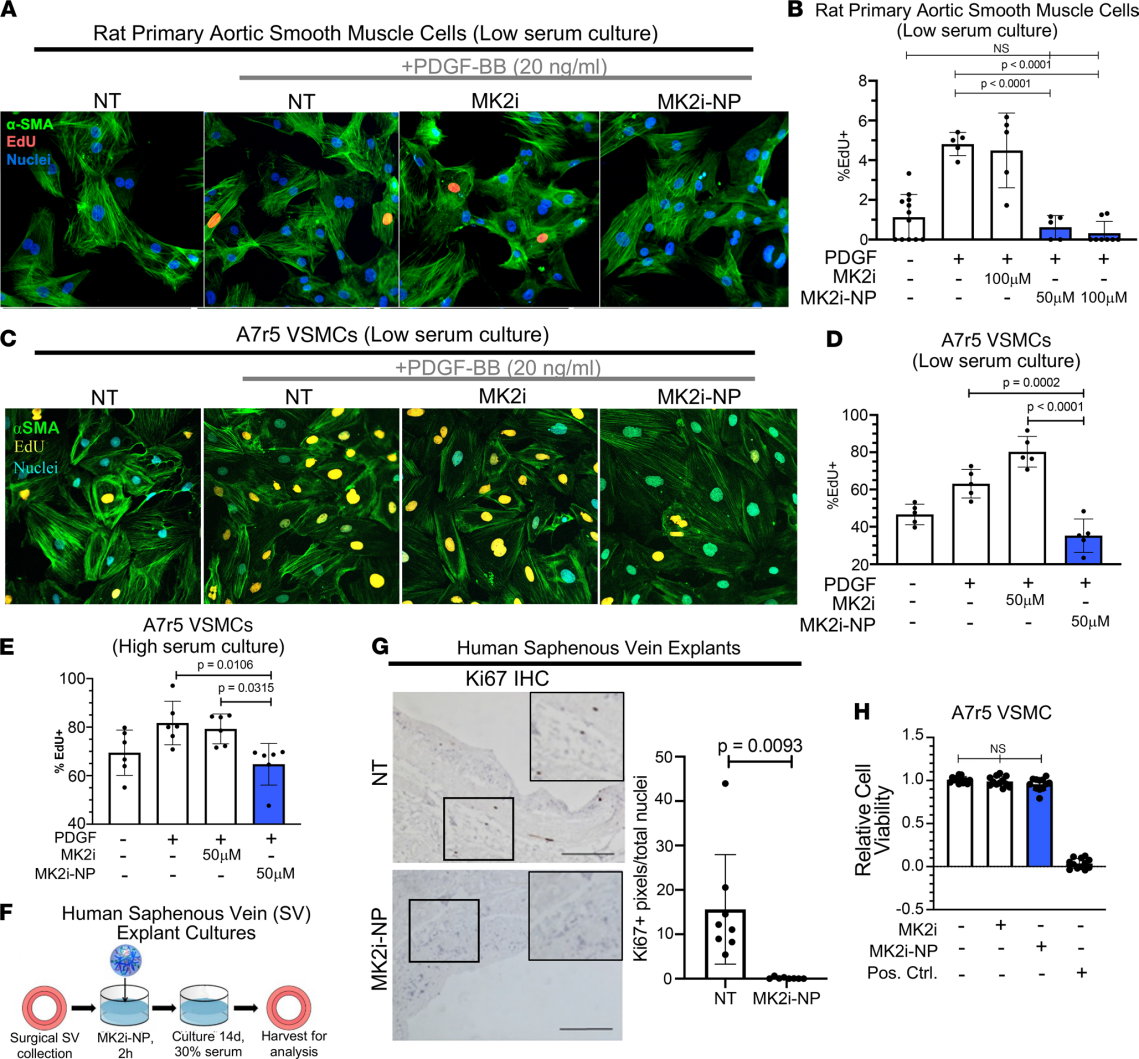
MK2i-NPs provide extended inhibition of proliferation in vitro and ex vivo without affecting viability. (**A**) Primary rat aortic smooth muscle cells (RASMCs) were quiesced overnight in 0.1% FBS media. RASMCs were then treated with 50 or 100 μM of MK2i or MK2i-NPs (50 μM MK2i, 2.5 μM PPAA) for 2 hours and subsequently incubated in media containing 20 ng/mL PDGF. After 24 hours, cellular proliferation was assessed through immunocytochemistry. (**B**) The percentage of EdU-positive nuclei was counted using *n* ≥ 5 images to get the percentage of proliferating cells per field of view for each treatment group. Significance was determined using a 1-way ANOVA with Tukey’s multiple-comparison test. (**C**) A7r5 cells were cultured in 2% serum media and treated with 50 μM MK2i or MK2i-NPs (50 μM MK2i, 2.5 μM PPAA) for 2 hours and subsequently incubated in media containing 20 ng/mL PDGF. After 24 hours, cellular proliferation was assessed through immunocytochemistry in (**D**). (**E**) A7r5 culture, MK2i treatment, and PDGF stimulation were repeated in 10% serum with the percentage of EdU-positive cells quantified 24 hours after PDGF treatment. (**F**) HSV rings were treated for 2 hours with either vehicle or 100 μM MK2i-NPs (100 μM MK2i, 12.5 μM PPAA) and then cultured for 14 days in 30% serum media, replacing media every other day. (**G**) HSV rings were stained for Ki67 and Ki67^+^ pixels per nuclei were assessed to determine relative proliferation levels in *n* = 8 representative images. Statistical significance was determined using Welch’s *t* test. Scale bars: 200 μm. (**H**) A7r5 cells were treated with 50 μM MK2i or MK2i-NPs (50 μM MK2i, 5 μM PPAA, lyophilized) for 2 hours and incubated for 24 hours before measuring viability with the resazurin salt assay (*n* = 12). Bleached cells were used as a positive control for the cytotoxicity measurement and 1-way ANOVA was used to analyze statistical significance.

Next, we examined the impact of MK2i-NPs on VSMC proliferation. Serum-depleted rat primary aortic SMCs (PA-SMCs) were treated with free MK2i or MK2i-NP^50:2.5Lyo^ for 2 hours, washed, and then stimulated 24 hours with PDGF, a known inducer of VSMC proliferation that is often upregulated in IH (37). PA-SMC proliferation, as measured by EdU incorporation (Figure 2A), was increased upon PDGF-BB treatment by more than 3.2-fold (Figure 2B and Supplemental Table 1). Although pretreatment for 2 hours with free MK2i did not significantly affect PDGF-induced proliferation, pretreatment with MK2i-NP^50:2.5Lyo^ remarkably decreased PA-SMC proliferation to levels seen in unstimulated PA-SMCs. Similarly, A7r5 VSMCs quiesced in 2% serum exhibited increased EdU incorporation at 24 hours after addition of PDGF (Figure 2, C and D) but not if cells were pretreated with MK2i-NP^50:2.5Lyo^. To determine the impact of MK2 inhibition on VSMC proliferation after the synthetic phenotype has been established, A7r5 cells were cultured in full serum to induce the synthetic phenotype switch, then subsequently treated 2 hours with MK2i-NPs, washed, then treated with PDGF for 24 hours, maintaining serum in cultures throughout. Even in the context of serum and PDGF signaling, cells treated with MK2i-NP displayed robust inhibition of EdU incorporation (Figure 2E). These findings were verified further using human saphenous vein (HSV) surgical remnants (Vanderbilt University Medical Center) treated 2 hours with MK2i-NP^100:2.5Lyo^ or vehicle. Specimens were washed, then cultured 14 days in 30% serum (Figure 2F). IHC detection of the proliferation marker Ki67 revealed that HSV explants treated only 2 hours with MK2i-NP^100:2.5Lyo^ harbored 99% fewer proliferating cells as compared with PBS-treated control HSV explants (Figure 2G). Importantly, cell viability of A7r5 cells was unaffected by MK2i-NP^50:2.5Lyo^ or free MK2i (Figure 2H and Supplemental Table 2). Previous studies have shown that PPAA alone has no significant effect on cell viability (31).

### MK2i-NP treatment blocks molecular markers of the synthetic phenotype in VSMCs.

**Figure 3:**
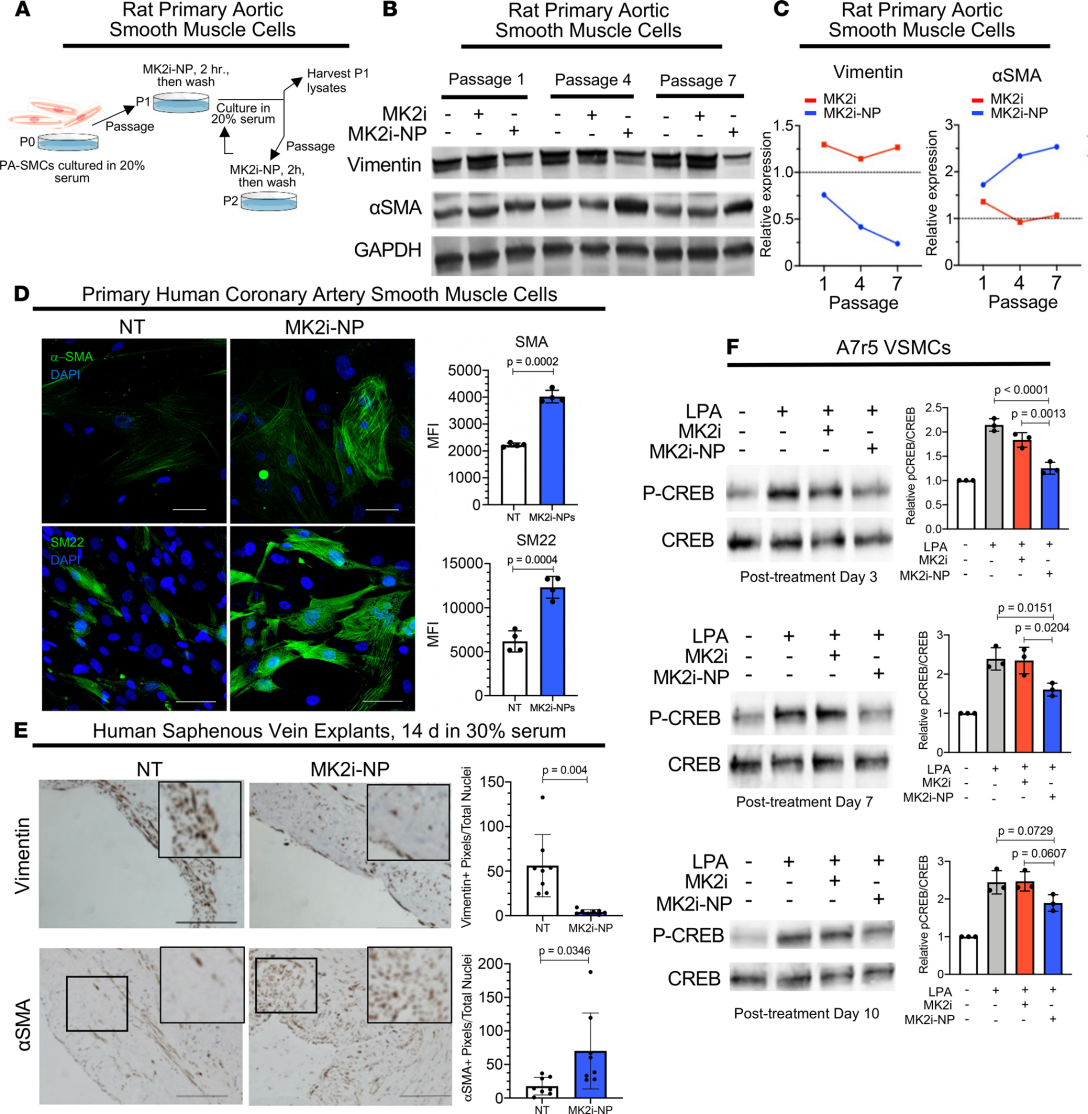
MK2i-NP treatment prevents phenotypic modulation of in vitro smooth muscle cells and ex vivo human saphenous veins while providing long-lasting pharmacodynamic effects. (**A**) Primary rat aortic SMCs were treated with 50 μM MK2i or MK2i-NPs (50 μM MK2i and 2.5 μM PPAA) for 2 hours at the time of passaging and then incubated in fresh high-serum medium (20% FBS). (**B**) The levels of α-SMA and vimentin were measured relative to GAPDH at passages 1, 4, and 7 with Western blots. The protein levels in the MK2i-NP–treated group relative to the no treatment group was plotted in (**C**). Measurements were taken on pooled RASMCs from 4 rats. (**D**) HCA-SMCs were cultured in 20% serum media and treated with MK2i-NPs at each passage up to passage 7. Levels of α-SMA and SM22 were analyzed with immunofluorescence. *n* = 4. Scale bars: 50 μm. Statistical significance was determined with Welch’s *t* test. (**E**) HSV rings were treated for 2 hours with either vehicle or 100 μM MK2i-NPs (100 μM MK2i, 12.5 μM PPAA). The samples were then cultured for 14 days in high-serum media (30% FBS), replacing media every other day. HSV rings were stained for vimentin and α-SMA to assess relative levels of synthetic and contractile phenotypes, respectively. The number of vimentin^+^ or α-SMA^+^ pixels per nuclei was plotted for *n* = 8 representative images. Statistical significance was determined using Welch’s *t* test. Scale bars: 200 μm. (**F**) A7r5 cells were treated with 50 μM MK2i or MK2i-NPs (50 μM MK2i and 2.5 μM PPAA) and cultured for 3, 7, or 10 days after treatment prior to LPA stimulation and protein harvesting for Western blot analysis of CREB phosphorylation, used as a pharmacodynamic biomarker for MK2 inhibition. *n* = 3. Statistics were determined using 1-way ANOVAs and Tukey’s multiple-comparison test.

Synthetic VSMCs, which contribute to the pathogenesis of IH, often exhibit diminished expression of contractile proteins like α-SMA but increased abundance of proteins involved in cell motility, like vimentin (38). To determine the impact of MK2 inhibition on maintenance of the synthetic VSMC phenotype, we cultured rat PA-SMCs in 20% serum to induce synthetic phenotype switching beginning at passage 0. PA-SMCs were treated with MK2i-NP^50:5Lyo^ for 2 hours upon each subsequent passage, then washed before continued culture in 20% serum (Figure 3A). Western blot analysis of cells collected at passages 1, 4, and 7 showed abundant vimentin expression in PBS-treated cells and in cells treated with free MK2i (Figure 3B). However, vimentin levels were decreased in cells treated with MK2i-NP^50:5Lyo^ at each passage, an observation that became increasingly pronounced at later passages (Figure 3C). Conversely, α-SMA expression was higher in cells treated with MK2i-NP^50:5Lyo^ at each passage, as compared with what was seen in cells treated with PBS or with free MK2i (Figure 3B). Again, the difference became more pronounced at later passages, with MK2i-NP^50:5Lyo^-treated cells maintaining more than 2.5 times α-SMA (Figure 3C). Additionally, a maintenance of contractile markers α-SMA and SM22 was seen in human primary coronary artery SMCs (PCA-SMCs) cultured out to passage 7 in high-serum conditions after MK2i-NP treatment (Figure 3D). Similarly, an increase in synthetic markers vimentin, osteopontin, and Fn-EDA was seen in untreated PCA-SMCs at passage 7 (Supplemental Figure 2).

These findings were verified using HSV surgical remnant cultures pretreated 2 hours with MK2i-NP^50:2.5Lyo^, then cultured 14 days in 30% serum, as described above (see Figure 2F). IHC analyses revealed abundant vimentin staining in HSV explants after 14 days of culture (Figure 3E), consistent with acquisition of synthetic VSMC traits. However, HSV explants treated with MK2i-NP^100:2.5Lyo^ displayed dramatically reduced vimentin levels at culture day 14. Conversely, pretreatment for 2 hours with MK2i-NP^100:2.5Lyo^ resulted in increased expression of the contractile marker α-SMA at culture day 14. Importantly, previous studies showed that in a similar HSV model, PPAA alone had no effect on neointima formation after 14 days (33). These studies showed that MK2i-NPs promoted contractile VSMC differentiation, reduced synthetic VSMC traits, and provided a durable 14-day effect after only a 2-hour treatment. To more thoroughly investigate the pharmacodynamic durability of MK2 inhibition by MK2i-NPs, we treated A7r5 VSMCs for 2 hours with MK2i-NP^50:2.5Lyo^ or free MK2i. The cells were then washed, cultured for 3–10 days, and treated with LPA to induce MK2-dependent CREB phosphorylation, which was confirmed by Western blot analysis (Figure 3F). Free MK2i diminished LPA-induced phosphorylated CREB (p-CREB) by approximately 30% at day 3 after treatment. However, MK2i-NP^50:2.5Lyo^ reduced LPA-induced p-CREB to an even greater extent at day 3 after treatment (80% reduction). By days 7 and 10, LPA-induced p-CREB remained high in cells treated with free MK2i or with LPA alone. In contrast, MK2i-NP^50:2.5Lyo^-treated cells displayed markedly diminished p-CREB levels at day 7 after treatment (45% reduced) and day 10 (38% reduced), although p-CREB diminution at day 10 only approached significance (*P* = 0.06) (full statistics in Supplemental Table 3). These results confirmed the enhanced potency and longevity of MK2i-NP bioactivity in cells up to 10 days after treatment, giving stronger and longer lasting inhibition relative to free MK2i, and confirmed that inhibition of MK2 signaling in synthetic VSMCs using MK2i-NP promoted features of contractile VSMC transdifferentiation.

### Broad gene expression changes correlating with contractile differentiation in VSMCs upon MK2 inhibition.

**Figure 4:**
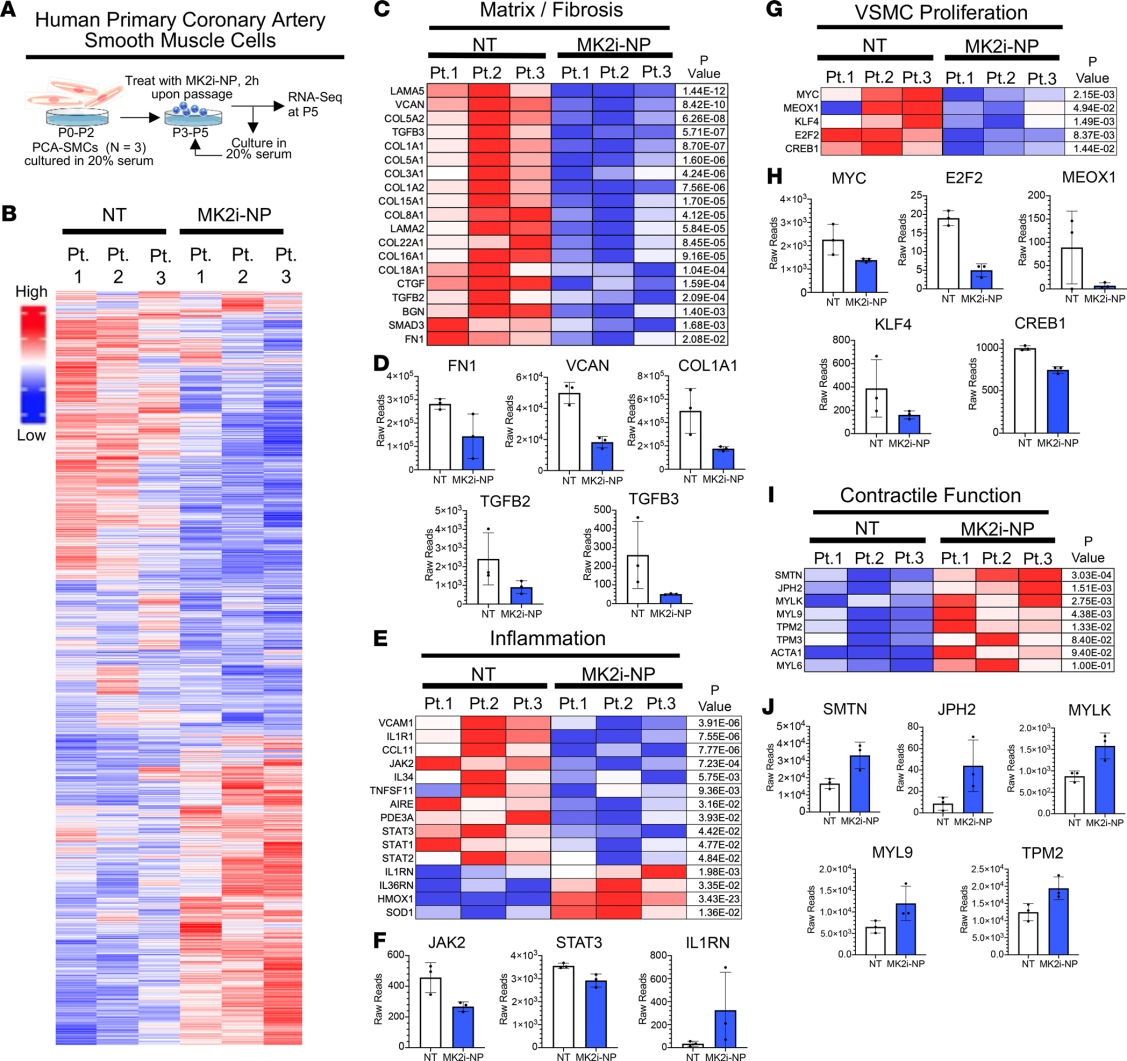
RNA-Seq analysis of primary HCA-SMCs after MK2i-NP treatment shows decreased expression of synthetic genes while maintaining expression of contractile genes. (**A**) Primary HCA-SMCs obtained from 3 patients were cultured out to P5 in 20% serum with either no treatment or 50 μM MK2i-NPs for 2 hours at each passage. RNA was harvested and sequenced to determine differences in genetic expression after MK2i-NP treatment. (**B**) Overall gene expression differences between treated and untreated cells for the 3 biological replicates. Relative expression levels for genes related to (**C**) fibrosis and ECM production, (**E**) inflammation, (**G**) proliferation, and (**I**) contractile machinery are plotted in a heatmap with raw reads for select genes from each plotted in **D**, **F**, **H**, and **J**, respectively.

We used RNA-Seq as an unbiased approach to assess the impact of MK2 inhibition on serum-induced contractile-to-synthetic phenotype switching in human VSMCs. For these experiments, PCA-SMCs harvested from 3 clinical donors were cultured in serum through passage 2. From passage 3 through passage 5, human PCA-SMCs were treated 2 hours with MK2i-NP^50:5Lyo^ or vehicle upon plating, washed, and then cultured in 20% serum until reaching 70% confluence (Figure 4A). The heatmap shown in Figure 4B revealed 1114 significantly upregulated and 1226 significantly downregulated genes in PCA-SMCs as a result of MK2 inhibition (Supplemental Data File 1). Importantly, several genes encoding ECM proteins (*FN*, *VCAN*, *LAMA2*, *LAMA5*, *COL1A1*, *COL3A1*) with known overexpression in IH (39, 40) were significantly downregulated in VSMCs treated with MK2i-NP (Figure 4C). Specifically, fibronectin (*FN*), a well-described synthetic VSMC marker and putative potentiator of the synthetic VSMC phenotype (17), was downregulated 51.6% in response to MK2i-NP treatment, as was expression of *TGFB2* and *TGFB3*, encoding 2 isoforms of TGF-β, a pleiotropic cytokine that directs FN expression (41) and promotes VSMC migration (42) (Figure 4, D and E). Notably, multiple factors of the TGF-β pathway were downregulated (Supplemental Table 4). Further, a panel of proinflammatory genes (*CCL11*, *IL34*, *AIRE*, *TNFSF11*, *JAK2*, *STAT1*, *STAT3*) was coordinately downregulated upon MK2 inhibition (Figure 4E), whereas antiinflammatory genes (e.g., *IL1RN*) were upregulated, demonstrating reversal of another molecular trait of synthetic VSMCs that drive dysregulated IH (Figure 4F and Supplemental Table 5). An additional feature of synthetic VSMCs is their enhanced proliferative state, driven by transcription factors encoded by *CREB1*, *KLF4*, *E2F2*, *MYC*, and *MEOX1* (28, 43–46), which were each significantly decreased in MK2i-NP^50:5Lyo^-treated cells (Figure 4, G and H). Furthermore, MK2i-NP^50:5Lyo^ treatment increased levels of contractile phenotype markers smoothelin (*SMTN*), tropomyosin (*TPN2*, *TPN3*), and myosin light chain (*MYL8*, *MYL9*, *MYLK*) (Figure 4, I and J). These results were supplemented by gene set enrichment analysis (GSEA) (Supplemental Figures 3 and 4) that showed MK2i-NP treatment was negatively correlated with the expression of several Hallmark and Kyoto Encyclopedia of Genes and Genomes (KEGG) gene sets associated with proliferation and inflammation. These results collectively demonstrated that MK2 inhibition successfully promoted VSMC transdifferentiation toward a contractile phenotype and away from the proliferative, proinflammatory, and ECM-producing phenotype of synthetic VSMCs.

### MK2i-NPs block neointima formation and VSMC synthetic phenotype switch in autologous vascular transplants in vivo.

**Figure 5:**
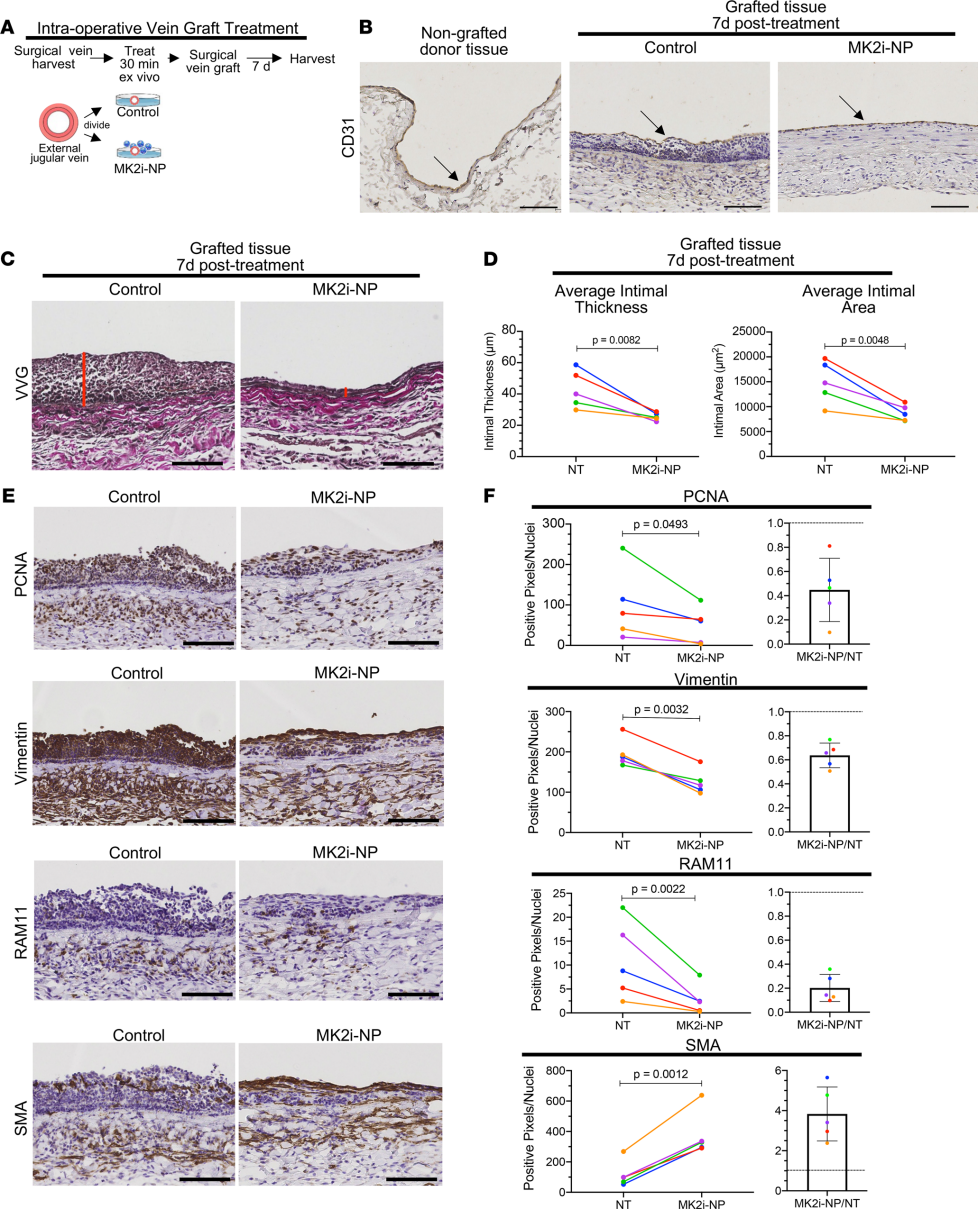
MK2i-NPs maintain the contractile VSMC phenotype while reducing expression of synthetic phenotype markers in rabbit external jugular vein grafted into carotid artery. (**A**) During surgery, rabbit EJVs were treated in PlasmaLyte with either lyophilized MK2i-NPs^50:5Lyo^ or a lactosucrose excipient control for 30 minutes and then grafted bilaterally into the rabbit carotid arteries. (**B**) After 7 days, the grafts were perfusion-fixed and stained for CD31 to assess endothelial coverage. Scale bars: 100 μm. (**C**) Verhoeff–van Gieson staining was done on graft sections to highlight the neointimal layer. (**D**) Neointimal thickness and cross-sectional area were quantified. (**E**) Vein graft sections were stained for PCNA, vimentin, RAM11, and α-SMA to show relative levels of proliferation, inflammation, and phenotype switching. Representative images of the area of largest neointima formation were taken from a pair of EJVs from the same rabbit. Scale bars: 100 μm. (**F**) Immunohistochemically stained sections of grafts were quantified using a color deconvolution plugin in ImageJ. The ratio of stain-positive pixels to number of nuclei was plotted as well as the ratio of nanopolyplex (NP) to no treatment (NT) for each of the 5 rabbits. Analysis was performed on images of 6 evenly spaced tissue sections from each graft, and the average for each graft was used in a paired analysis. Each line represents paired treated and untreated contralateral grafts within the same rabbit. Statistical significance was determined with a 2-sided ratio paired *t* test.

Given that MK2 inhibition blocks key traits of synthetic VSMCs, including proliferation and production of ECM and proinflammatory cytokines, and given the durable efficacy of a single MK2i-NP dose, we evaluated MK2-mediated VSMC phenotype switching in the context of surgical vascular grafting in vivo. These studies used rabbit external jugular vein (EJV) grafting, where grafts were ex vivo topically treated intraoperatively with MK2i-NP prior to grafting onto rabbit carotid arteries. The grafted EJV segments were surgically collected and divided. One segment was briefly (30 minutes) treated ex vivo with MK2i-NP^50:5Lyo^ in PlasmaLyte (Figure 5A), and the other was treated with 30 mM lactosucrose in PlasmaLyte, followed by surgical anastomosis with carotid artery segments. The grafts were analyzed 7 days after surgery to capture the period of highest cellular proliferation and inflammation (47). IHC staining for the endothelial cell (EC) marker CD31 (Figure 5B and Supplemental Figure 5) showed that graft EC coverage was continuous in samples treated intraoperatively with control solution or with MK2i-NP^50:5Lyo^, similar to what was seen in the donor EJV tissue prior to grafting. However, Verhoeff–van Gieson staining, which was used to visualize vessel neointima (Figure 5C), revealed substantially reduced neointimal thickness and area in surgical vein grafts treated intraoperatively with MK2i-NPs (*n* = 5, Figure 5D). Proliferating cell nuclear antigen (PCNA) staining revealed abundant cellular proliferation within the vascular neointimal layer of control-treated samples (Figure 5E). However, MK2i-NP^50:5Lyo^ treatment decreased proliferation in 5 of 5 surgical samples by an average of nearly 55% (Figure 5F). Vimentin, a molecular marker of synthetic VSMCs, was markedly reduced (36% reduction) in 100% of MK2i-NP^50:5Lyo^-treated vascular grafts (Figure 5, E and F). Macrophage accumulation, used as a proxy for vascular inflammation, was examined using IHC for RAM11, which was readily detected in vehicle-treated grafts. However, we found starkly reduced macrophage accumulation (80% reduction) in 5 of 5 vascular grafts treated intraoperatively with MK2i-NP^50:5Lyo^, demonstrating in vivo that multiple markers of synthetic VSMC, each of which contributes to IH pathophysiology, were diminished upon intraoperative treatment of grafted vascular tissue with MK2i-NP. Conversely, the contractile VSMC marker α-SMA was increased 3.8-fold in MK2i-NP^50:5Ly^–treated grafts over what was seen in vehicle-treated grafts (Figure 5C). Together, these data demonstrated the important role of MK2 signaling in driving the contractile-to-synthetic phenotype switch in VSMCs and showed that treatment with MK2i-NPs maintained the contractile VSMC phenotype to minimize vascular graft IH.

## Discussion

Here, we demonstrated durable and potent MK2 inhibition as a means of blocking contractile-to-synthetic VSMC phenotypic switching after vascular intervention. Given the relationship between synthetic VSMCs and IH and observations that IH is a driving cause of vessel occlusion after vascular intervention (1–3), these findings support the hypothesis that pharmacological MK2 inhibition may prevent IH and would thus maintain patency and integrity of vascular grafts and sites of vascular angioplasty. Our data showed that intraoperative MK2i-NP delivery to donor vessels ex vivo immediately prior to grafting blocked VSMC synthetic differentiation (Figure 5), prevented IH, and preserved vessel patency (Figure 4).

Synthetic VSMCs exhibit several distinguishing traits, including enhanced proliferation, increased expression of proinflammatory cytokines, and augmented production of ECM proteins. While each of these synthetic VSMC traits contributes to pathologic IH (13, 48, 49), clinical trials in IH have focused on drugs aimed at blocking VSMC proliferation but without blockade of the other traits of synthetic VSMCs. For example, the E2F decoy edifoligide inhibits the ability of E2F to interact with its target genes, inhibiting its proliferative effects (50). However, the E2F decoy failed to show improvement in preventing vein graft failure in clinical trials (12). Currently approved strategies for combating IH use either chemotherapy or mTOR inhibition embedded within drug-eluting balloons and stents to block proliferation by VSMCs, and to a lesser extent, to minimize inflammation. However, neither of these drugs fully targets all VSMC trains during IH, including VSMC migration to the intima, neointimal ECM deposition by VSMCs, and proinflammatory cytokine expression by VSMCs. In contrast, we have shown here that inhibition of VSMC contractile-to-synthetic phenotype switching prevents the pathological accumulation of disease-associated synthetic VSMCs, thus comprehensively targeting the VSMC traits that initiate and exacerbate IH.

Importantly, we have established the serine-threonine kinase MK2 as a molecular switch that allows VSMCs to toggle between contractile and synthetic phenotypes, such that MK2 activity drives VSMCs toward a synthetic phenotype, while MK2 inhibition reverses dedifferentiation to preserve a contractile phenotype. MK2 is directly phosphorylated by stress-activated p38 MAPK (22), a kinase that activates a multitude of pathways involved in inflammation, cell migration, and cell proliferation. Notably, p38 MAPK is activated in VSMCs, particularly in the context of IH. However, pharmacological p38 MAPK inhibitors failed clinical trials because of associated toxicities and increased risk of certain cancers (51, 52), perhaps a result of the widespread expression/activity of p38 MAPK and its wide network of substrates. These observations motivated the search for druggable pathways downstream of p38 MAPK in VSMCs and IH. As a p38 MAPK substrate, MK2 phosphorylates multiple proteins that drive the VSMC synthetic phenotype, including CREB and HSP27 (28, 29). Each of these factors independently has a known role in VSMC-mediated IH, suggesting that MK2 may be a valid molecular target to combat IH. We showed here that MK2 inhibition blocked phosphorylation of CREB (Figure 2) and diminished expression of KLF4, E2F2, MEOX, and other drivers of the synthetic VSMC phenotype (Figure 3), further minimizing the VSMC phenotypic switch. Additionally, delivery of the MK2 inhibitor to vessels ex vivo using our polymeric NP formulation minimized systemic exposure to the MK2 inhibitor, while providing potent and stable MK2 inhibition within the surgically targeted tissue (Figure 5).

Discovery of small molecular weight MK2 kinase inhibitors has been met with challenges (53). Here, we used a peptide that binds to MK2, blocking its catalytic activity (54). Intracellular peptide drug delivery has its own challenges, including short circulation half-lives and endosomal entrapment, which permits extracellular recycling and/or lysosomal degradation (55, 56). We have addressed the first issue using an intraoperative topical treatment of explanted tissue, a strategy shown to be feasible in various animal and clinical trials (12). To enable endosome escape of the inhibitory peptide, we packaged the MK2i peptide with PPAA, a strategy that also potentiates the level of peptide uptake (Figure 1). The alkyl propyl side chains of PPAA coat cell membranes through hydrophobic interactions, while the anionic carboxylate groups bait cationic peptides to the cell surface to promote internalization at physiological pH (31). Within endosomes, however, carboxylic acid protonation of PPAA increases its hydrophobicity, causing endosomal membrane disruption and cytosolic delivery (31). Secondarily, endosome disruption extends intracellular retention of the inhibitory peptide (33, 57). Optimization of intracellular peptide retention is of particular importance in the vascular graft treatment setting used herein, in which a single prophylactic treatment was given intraoperatively to provide a therapeutic benefit throughout the graft healing process. Increased cellular uptake and retention make MK2i-NPs the ideal treatment strategy over previously explored methods, such as a heparin-functionalized graft that elutes free MK2i peptide over time (58), which does not have the intracellular bioavailability benefits that come with PPAA complexation. A previous vascular graft study showed the greatest VSMC proliferation and inflammation in the first week after surgery, which waned thereafter (47), highlighting a critical window through which intraoperative vascular treatments must be retained as the vascular graft heals. Importantly, we found that a single brief treatment with MK2i-NPs reduced LPA-induced CREB phosphorylation downstream of MK2 through at least 10 days (Figure 2), supporting the feasibility of this approach.

MK2i-NPs are a flexible and potent alternative to drug-eluting stents or balloons for delivery of compounds targeting IH. One important study described surface-based delivery of peptide from a synthetic polymer film that could potentially be developed as a vascular graft material (58). This biomaterial was functionalized with anionic heparin to which the cationic MK2i peptide was electrostatically adhered, allowing slow release of free MK2i peptide from the surface when applied in cultures. In contrast, we describe here an aqueous formulation of NPs that allows cells or tissues to be treated ex vivo, eliminating the need for synthetic graft implantation. Importantly, MK2i-NPs enhance activity of MK2i relative to treatment with the free peptide alone through promoting higher cellular uptake and facilitating endosome disruption and consequent cytoplasmic bioavailability and retention.

In summary, we have shown that MK2 plays a key role in contractile-to-synthetic VSMC phenotype switching, such that MK2 inhibition prevented VSMC synthetic dedifferentiation as well as synthetic VSMC-driven IH and vascular graft failure. Future studies will be required to assess long-term impact of intraoperative vascular treatment with MK2i-NPs on VSMC phenotype and clinical outcomes. It will be particularly important to also advance future studies into larger scale preclinical models. While we have defined MK2 as a molecular switch regulating phenotype conversion in VSMCs, it will also be enlightening to determine which specific downstream targets of MK2 regulate phenotype switching and to elucidate any phosphatases that may downregulate this signaling pathway. The continued study of prophylactic MK2i-NP treatment for postprocedural IH is warranted based on these promising findings.

## Methods

### Materials.

An MK2 inhibitory peptide with the sequence YARAAARQARAKALARQLGVAA was synthesized by and purchased from EZBioLab with a purity of 95% or greater as determined by mass spectrometry. The Click-iT EdU kit was obtained from Thermo Fisher Scientific. PDGF-BB and LPA were purchased from MilliporeSigma. Antibodies against PCNA (catalog ab912), Ki67 (catalog ab1580), vimentin (catalog ab28028), α-SMA (catalog ab781), SM22 (catalog ab14106), and osteopontin (catalog ab166709) were purchased from Abcam. CREB (catalog 9104), p-CREB (catalog 9198), and additional vimentin (catalog D21H3, 5741) and α-SMA (catalog 19245) antibodies were purchased from Cell Signaling Technology. RAM11 (catalog M0633) antibodies were purchased from DAKO/Agilent. GAPDH (catalog MAB374) and Fn-EDA (catalog F6140) antibodies were purchased from MilliporeSigma. CD31 (catalog AM50226PU-S) antibodies were purchased from OriGene. For IHC, primary antibodies were used at 1:100 (RAM11), 1:150 (vimentin), 1:500 (α-SMA), or 1:25 (CD31) dilutions, and secondary antibodies (catalog BA-9200) were purchased from Vector Laboratories and used at a 1:2000 dilution. For immunocytochemistry, primary and secondary antibodies were used at a 1:500 dilution.

### MK2i-NP synthesis.

PPAA was synthesized via bulk RAFT polymerization, purified, and characterized as previously described (31, 33). Briefly, the 2-propylacrylic acid (2-PAA) monomer was synthesized according to methods developed by Ferrito et al. (59) The chain transfer agent (CTA) 4-cyano-4-(ethylsulfanylthiocarbonyl) sulfanylpentanoic acid was synthesized as previously described (60). The monomer was mixed with 2,2′-azo-bis-isobutyrylnitrile (AIBN) recrystallized from dioxane as an initiator and the CTA with a molar ratio of CTA/AIBN of 1:1, and the 2-PAA/CTA ratio was set to achieve a molecular weight of 25,000 g/mol at full conversion. A magnetic stir bar was added to the reaction vessel, and the mixture was subjected to 3 freeze/vacuum/thaw cycles, purged with nitrogen for 30 minutes, and subsequently maintained under a nitrogen atmosphere for the duration of the polymerization reaction. The polymerization reaction was initiated by submerging the reaction vessel in a 70°C oil bath to activate the free radical initiator AIBN. The polymerization reaction was monitored for 48 hours until the reaction mixture became highly viscous. The resulting polymer was dissolved in dimethylformamide, precipitated into cold diethyl ether 5 times, and dried overnight under vacuum. PPAA molecular weight and polydispersity were determined using gel permeation chromatography (Agilent 1200 series GPC system with an in-line mini-DAWN T-rex light scattering detector, variable wavelength detector, and refractive index detector) and analyzed using Astra V software (Wyatt Technology) calibrated using PMMA and PEG standards (Agilent). Purity, composition, and molecular weights were further verified using proton nuclear magnetic resonance.

MK2i peptide was dissolved in PBS (pH 8) to obtain a stock solution. PPAA was dissolved in 1 M NaOH and diluted into phosphate buffer (pH 8). A pH of 8 was chosen as a median between the acid dissociation constants of the carboxylate anion on PPAA and the primary and secondary amines on the MK2i peptide to facilitate electrostatic complexation. MK2i and PPAA solutions were mixed in phosphate buffer at concentrations of 500 μM and 25 or 50 μM, respectively, to form a 10× stock of MK2i-NPs. The resulting polyplexes were syringe-filtered through 0.45 hydrophilic polytetrafluoroethylene (PTFE) filters from Millex.

### MK2i-NP lyophilization.

To lyophilize MK2i-NPs for long-term storage, a previously described method was used (34) with alteration in peptide and polymer relative quantities based on previous studies (31). Briefly, MK2i-NPs were formulated at concentrations of 500 μM MK2i and 50 μM PPAA to form a 10× stock with 300 mM lactosucrose added as a lyoprotectant to the NP solution. The NPs were then syringe-filtered through a 0.45 μM PTFE filter and separated into 200 μL aliquots. They were then frozen to –80°C for 24 hours and lyophilized. NPs were reconstituted for 30 minutes before use in in vivo studies (detailed below) by addition of an appropriate amount of sterile water for injection to achieve the desired concentration for dosing.

### Cell culture.

A7r5 rat aortic SMCs were obtained from American Type Culture Collection (ATCC). A7r5 cells were cultured in high glucose DMEM supplemented with 10% FBS and 1% penicillin/streptomycin (P/S). Cells were maintained in 37°C and 5% CO_2_ environment. Only cells from passages 3–8 were used in experiments. For LPA treatments, A7r5 cells were quiesced in low-glucose (1 g/L) and low-FBS (0.1% FBS) media for 24 hours prior to LPA treatment. The treatment was then removed, and the cells were either immediately stimulated with 30 μM LPA for 30 minutes or cultured for an additional 1, 3, 7, or 10 days in low-glucose, low-serum media prior to LPA stimulation. Primary rat aortic SMCs were collected from 4 male Wistar rats (The Jackson Laboratory), endothelium was removed by swabbing, and the remaining tunica media was dissociated (Miltenyi Biotec tissue dissociation kit) according to the manufacturer’s instructions for 40 minutes. Subsequently, cell suspensions were forced through a 70 μm strainer washed, pooled, and cultured in a T-75 flask in DMEM with 20% FBS and 1% P/S until 80%–90% confluent. Primary human coronary artery SMCs from 3 human donors were obtained from ATCC (PCS-100-021). Human coronary artery SMCs were cultured using vascular cell basal medium (ATCC, PCS-100-030) supplemented with a VSMC growth kit (ATCC, PCS-100-042) and 20% serum to induce phenotype switching.

### Analysis of MK2i uptake with flow cytometry.

A7r5 cells were seeded into a 96-well plate at 5000 cells per well in DMEM supplemented with 10% serum and 1% P/S and allowed to adhere for 48 hours. MK2i peptide was conjugated with Alexa Fluor 488 (Thermo Fisher Scientific) according to the manufacturer’s instructions, and MK2i-NPs were made with the fluorescent peptide and lyophilized. Cells were treated with 50 μM MK2i-488 or 50 μM reconstituted MK2i-488-NPs (50:5_lyo_) in OptiMEM (Gibco) with 1% serum and incubated for 2 hours before treatment removal. For fluorescence imaging, cells were washed with 1% serum OptiMEM before being replaced with fresh 1% serum OptiMEM and imaged on a Nikon Eclipse Ti inverted fluorescence microscope. For flow cytometry, the treated cells were collected by trypsinization, then analyzed with a Guava easyCyte HT, and the resulting data were processed with FlowJo version 10.

### Gal8 recruitment assay in A7r5 cells.

Gal8^YFP^ measurements were performed as previously described (36) utilizing A7r5 cells stably transduced with a lentiviral Gal8-YFP expression vector. A7r5-Gal8^YFP^ cells were seeded in a 96-well plate at 2500 cells per well in DMEM supplemented with 10% serum and 1% P/S and allowed to adhere for 48 hours. Cells were treated with 50 μM MK2i or 50 μM MK2i-NPs (50:5_lyo_) in OptiMEM with 1% serum and incubated for 15 minutes before being imaged on a Nikon Eclipse Ti inverted fluorescence microscope using a 20× objective. Cells imaged 3 hours after treatment were used for quantification of endosomal disruption in MATLAB using a previously described method (36).

### Cell viability assay.

A7r5 cells were seeded into a 96-well plate at 5000 cells per well in DMEM supplemented with 10% serum and 1% P/S and allowed to adhere for 48 hours. Cells were treated with 50 μM MK2i or 50 μM MK2i-NPs (50:5_lyo_) in OptiMEM with 1% serum. Treatments were removed after 2 hours, and the media were replaced with 10% serum DMEM containing 25 μg/mL resazurin salt. Plate absorbance was read every 5 minutes on a TECAN Infinite M1000 Pro plate reader, and the difference between the baseline measurement and the average of the 5 values at the peak of the kinetic curve was quantified for each well.

### Western blot analysis.

Cells and tissues were lysed in RIPA buffer with 1:200 protease inhibitor cocktail (MilliporeSigma) and phosphatase inhibitor I and II cocktails (MilliporeSigma) on ice for 1 hour, centrifuged at 10,000*g* for 15 minutes at 4°C. Protein concentration was quantitated by BCA assay (Pierce). Protein (20 μg) was resolved on 10% SDS-PAGE gels and transferred to PVDF membranes. Blots were incubated in primary and secondary antibodies for 1 hour each at room temperature, washed after each, and imaged (LI-COR Odyssey).

### HSV ex vivo assay and IHC.

Upon approval by Vanderbilt University Medical Center’s IRB, deidentified, discarded segments of HSV were collected from consenting patients undergoing coronary or peripheral vascular bypass surgeries. After surgical resection, HSV segments were stored in buffered salt solution until the end of the surgical procedure, at which time they were placed in cold transplant harvest buffer (100 mM potassium lactobionate, 25 mM KH_2_PO_4_, 5 mM MgSO_4_, 30 mM raffinose, 5 mM adenosine, 3 mM glutathione, 1 mM allopurinol, 50 g/L hydroxyethyl starch, pH 7.4). Damaged portions were not used for experiments. All HSV segments were used within 24 hours of harvest. Utilizing sterile technique in a sterile culture hood, the HSV segments were transferred into a 60 mm petri dish. The end of each segment (0.5 mm) was removed with a blade, and excess adventitial tissue and adipose tissue were removed with minimal manipulation. Segments were then cut into consecutive rings with an approximate width of 1.0–2.0 mm.

HSV rings were treated with 100 μM MK2i-NPs (100 μM MK2i, 12.5 μM PPAA) and then cultured for 14 days in high-serum RPMI 1640 (Gibco) (30% FBS to accelerate neointimal growth), 1% l-glutamine, and 1% P/S at 37°C in 5% CO_2_. After culture, vein segments were fixed in 0.5 mL of 10% neutral buffered formalin at 37°C for 30 minutes and embedded in paraffin. Transverse 5 μm sections were cut from the middle of the rings and stained for DAPI and Ki67, α-SMA, or vimentin by the Tissue Pathology Shared Resource Core at Vanderbilt University Medical Center. The samples were then imaged on a Nikon Eclipse Ti inverted fluorescence microscope. Intimal layers were selected in ImageJ (NIH) by selecting tissue within the internal and external elastic lamina, and positive staining was quantified using a previously described color deconvolution method (61). Positive staining was normalized to the number of intimal cell nuclei. Four images per patient from 2 donors to account for patient variability were analyzed for each treatment group.

### Primary human cell culture and RNA-Seq.

Primary HCA-SMCs were cultured out to passage 5 in 20% serum in parallel treatments with control vehicle or MK2i-NPs. Total RNA was extracted using a Qiagen RNeasy Mini kit (74104) and purity was assessed using a NanoDrop One. Total RNA was sent to Novogene for library preparation, sequencing, and analysis.

### Primary human cell immunocytochemistry.

Primary HCA-SMCs were cultured out to passage 7 in 20% serum and were either left untreated or treated with 50 μM MK2i-NPs (50:5_lyo_) for 2 hours at each passage. The cells were then fixed with 4% paraformaldehyde for 10 minutes, permeabilized with PBS containing 0.5% Triton X-100, and blocked with PBS containing 0.1% Triton X-100 and 5% FBS. The cells were washed 3 times with PBS between each step. The cells were then incubated with the primary antibodies overnight at 4°C and then treated with the secondary antibody and NucBlue for 1 hour at room temperature. The cells were imaged on a Nikon Eclipse Ti inverted fluorescence microscope. Composites of 9 images from each well were made and analyzed for MFI using a previously developed MATLAB script (36).

### Click-iT EdU assay.

Cells seeded on 8-well coverslips were treated as indicated and supplemented with 20 μM EdU for the final 24 hours of culture. Cells were fixed with 3.7% formaldehyde, permeabilized with 0.5% Triton X-100, blocked with 3% BSA in PBS, and incubated with 250 μL Click-iT plus reaction cocktail, followed by incubation with α-SMA antibody overnight at 4°C and fluorescent secondary antibody for 1 hour. Cells were counterstained with Hoechst (1:2000 dilution) and mounted with ProLong Gold Antifade (Thermo Fisher Scientific). Cells were imaged on a Nikon Eclipse Ti inverted fluorescence microscope.

### In vivo rabbit vein graft model.

Male New Zealand white rabbits (3.0 to 3.5 kg) were anesthetized through an intramuscular injection with ketamine hydrochloride (1.4 mg/kg) and xylazine (0.2 mg/kg). Anesthesia was maintained with endotracheal intubation and inhaled isoflurane (2.0% to 5.0%). A high-dose i.v. heparin bolus (250 U/kg) was administered immediately before carotid cross clamp. The operative procedure was performed with aseptic technique under ×2.5 optical magnification.

The rabbit bilateral jugular vein graft model was performed as previously described (33). During surgery, the rabbit’s EJV was removed and cuffs were inserted on either end. Meanwhile, lyophilized MK2i-NPs and lactosucrose controls were reconstituted for 30 minutes in sterile water. After reconstitution, the treatments were added to PlasmaLyte to achieve a final volume of 2 mL and a final concentration of 50 μM for the MK2i-NPs. Once excised, contralateral EJVs were given anastomotic cuffs on either end according to a previous method (62). The cuffed EJVs were soaked for at least 30 minutes in the MK2i-NP treatment or lactosucrose control, with the treated side being randomly determined. The carotid artery lumen was then cut open with a 2.0 cm arteriotomy, and the cuffed veins were inserted. Seven days after surgery, the rabbits were euthanized, and vein grafts were perfusion-fixed in situ with 10% neutral buffered formalin. The EJVs were removed, and 5 mm sections from the middle of the graft were cut and stored in 10% neutral buffered formalin for later histology.

### Rabbit histological quantification.

IHC was performed by the Vanderbilt University Medical Center Translational Pathology Shared Resource. First, 5 mm sections were formalin-fixed and paraffin-embedded. Sections (5 μm) were stained for PCNA, RAM11, α-SMA, and vimentin. The slides were imaged by the Vanderbilt University Medical Center Digital Histology Shared Resource and quantitated using ImageJ (NIH) using a previously described color deconvolution method (61). Sections from the midregion of each graft were analyzed, and the average ratio of positive pixels to number of nuclei was quantified for each section using 6 evenly spaced images. The images were taken starting at the area of most significant neointimal growth and moving evenly around the ring section. The average of the 6 images was used as the value for that rabbit in subsequent analyses.

### Data availability.

RNA-Seq data are available in NCBI’s Gene Expression Omnibus database (GEO GSE165715).

### Statistics.

Statistical significance for experiments with more than 2 groups was determined using 1-way ANOVA tests followed by Tukey’s post hoc test. For the in vivo rabbit study, 2-sided ratio paired *t* tests were used to determine significance between the treated and control groups. For HSV marker and immunocytochemistry quantification, Welch’s 2-tailed *t* test was used. Significance was accepted within a normal based 95% confidence limit (α = 0.05). Analyses were done with GraphPad Prism 8 software. Results are presented as arithmetic mean ± SD graphically with *P* values, as indicated in the figures or figure legends. *P* values of less than 0.05 were considered significant.

### Study approval.

All animal studies were approved by the Vanderbilt University IACUC and conformed to the *Guide for the Care and Use of Laboratory Animals* (National Academies Press, 2011). Use of HSV samples was approved by Vanderbilt University Medical Center’s IRB. Samples were collected from patients undergoing coronary or peripheral vascular bypass surgeries from whom written informed consent was obtained.

## Author contributions

JWT assisted with uptake characterization, the in vivo rabbit study, image analysis, and RNA-Seq preparation and analysis and wrote the manuscript. BCE performed CREB pharmacodynamics, PDGF proliferation, primary rat isolation and phenotype assessment, and HSV studies. BW performed Western blots and assisted with primary RASMC isolation and cell culture. JCF and MEP performed rabbit surgeries. JMC assisted with PDGF proliferation study and image analysis. RSC assisted with design and quantification of in vitro studies and manuscript editing. CMB and CLD conceived of and oversaw all studies. JWT and BCE share the first author position, with JWT being given the first order position based on his contributions in writing the manuscript.

## Supplementary Material

Supplemental data

Supplemental data set 1

## Version 1 10/08/2021

Electronic publication
