# Supplementary material for: Therapeutic MK2 inhibition blocks pathological vascular smooth muscle cell phenotype switch
Source: JCI Insight. 2021 Oct 8;6(19):e142339. doi: 10.1172/jci.insight.142339 (PMC8525639; doi:10.1172/jci.insight.142339)
Supplement: Supplemental data [file jciinsight-6-142339-s134.pdf]

Supplementary Information for

Therapeutic MK2 Inhibition Blocks Pathological Vascular Smooth Muscle Cell Phenotype Switch.

J. William Tierney, Brian C. Evans, Joyce Cheung-Flynn, Bo Wang, Juan M. Colazo, Monica E. Polcz, Rebecca S. Cook, Colleen M. Brophy, Craig L. Duvall

Corresponding author: Craig Duvall
Email: craig.duvall@vanderbilt.edu

This PDF file includes:

Figures S1-S5
Tables S1-S5

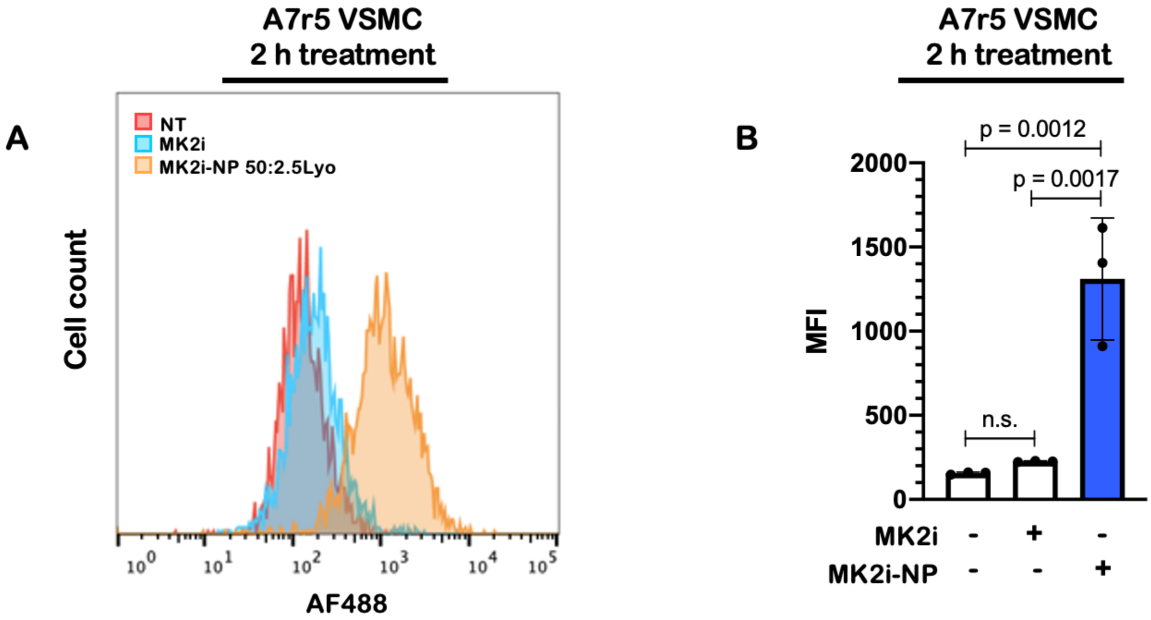

Figure S1. A7r5 cells were treated 2 hours with AlexFlour 488 (AF488)-labeled MK2i (50 μM), which was delivered as free-MK2i, or as MK2i-NP$^{50:2.5Lyo}$. Flow cytometric analysis was used to detect AF488 in cells. A. Representative histogram of flow cytometry experiments. B. Quantitation of the mean fluorescence intensity (MFI) per cell. Values shown are the average of (N = 3) experiments, ± S.D. P-values are derived from statistical analysis using one-way ANOVA with Tukey's repeated measures to compare individual groups.

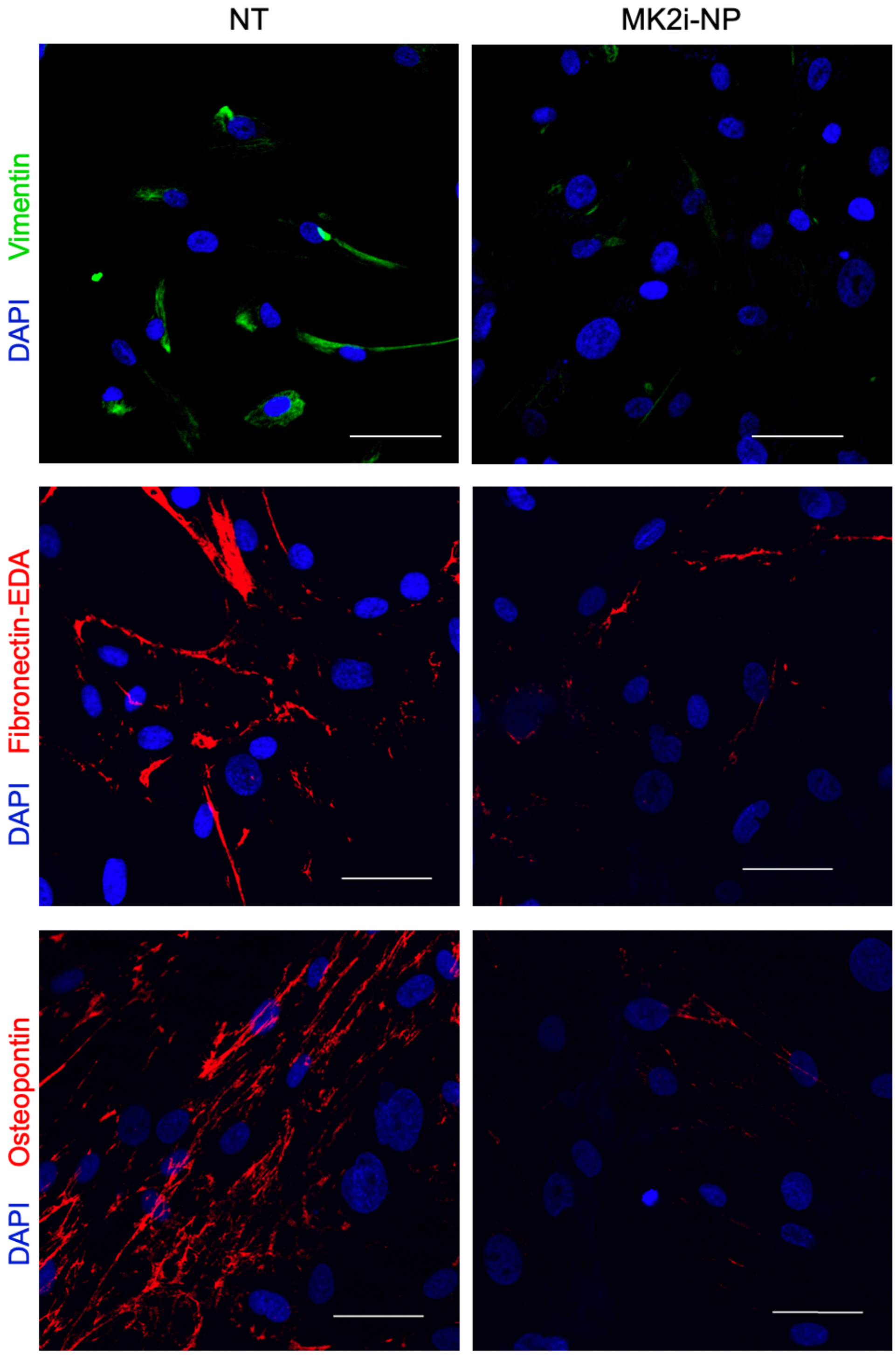

Figure S2. Immunocytochemistry shows reduction of multiple synthetic phenotype markers after MK2i-NP treatment. Primary human coronary artery smooth muscle cells were cultured out to P7 in 20% serum. Cells were either left untreated or treated with 50 μM MK2i-NPs for 2 hours at each passage. Scale bars are 50μm.

Hallmarks Gene Set Enrichment Analysis

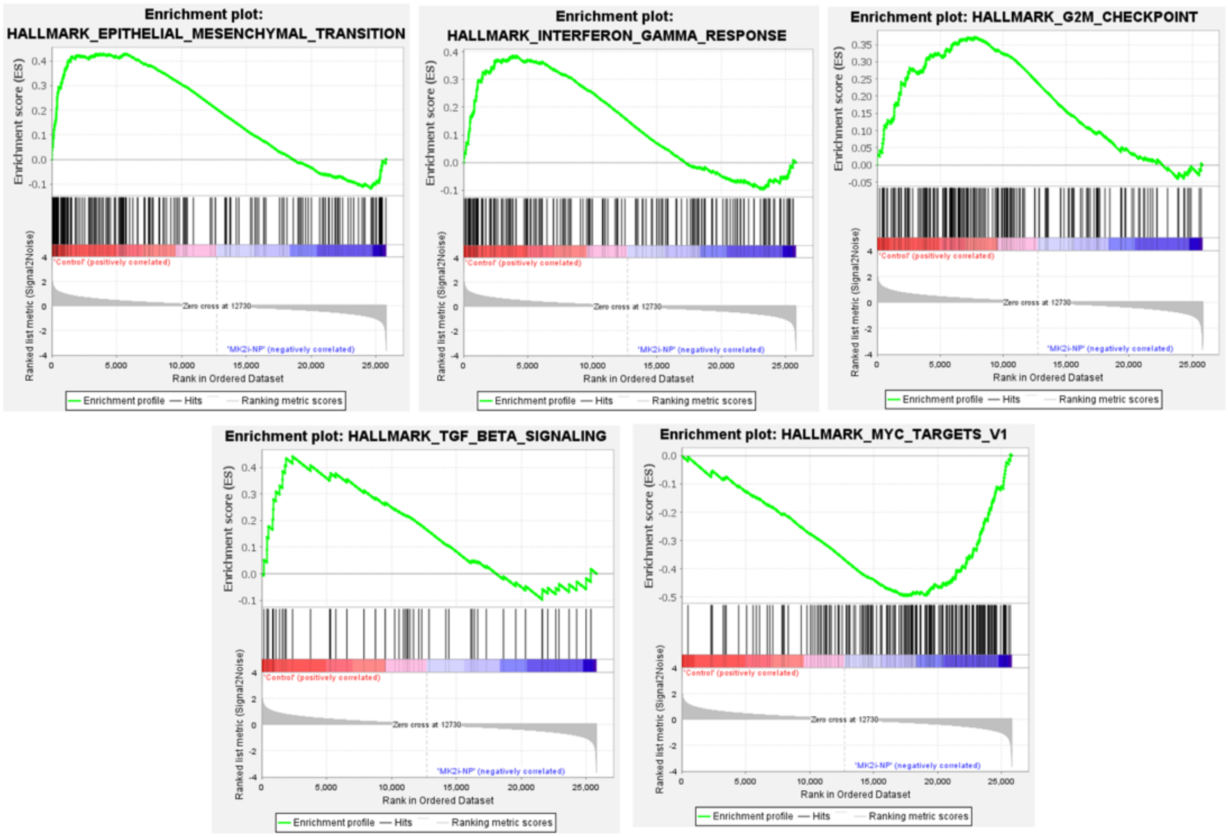

Figure S3. Significantly altered genes identified within RNA-Seq data of human primary coronary artery smooth muscle cells harvested from clinical samples treated with or without MK2i-NP were (N = 3 per group) were queried by Gene Set Enrichment Analysis using GSEA Software 4.1.0 (Broad Institute) for enrichment within the Hallmarks Gene Set (h.all.v7.3). Graphs were selected among the gene sets that were significantly correlated with altered genes in MK2i-NP-treated cells.

KEGG Pathway Gene Set Enrichment Analysis

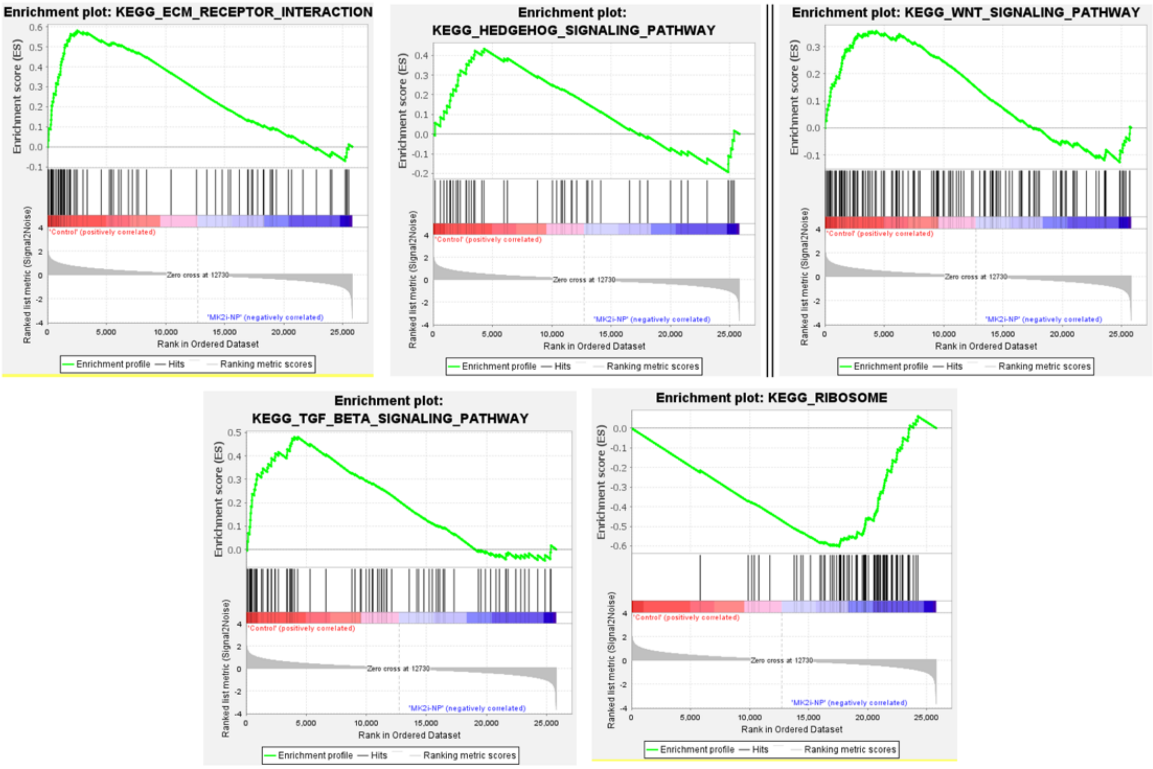

Figure S4. Significantly altered genes identified within RNA-Seq data of human primary coronary artery smooth muscle cells harvested from clinical samples treated with or without MK2i-NP were (N = 3 per group) were queried by Gene Set Enrichment Analysis using GSEA Software 4.1.0 (Broad Institute) for enrichment within the KEGG Pathway Gene Set (c2.cp.kegg.v7.1). Graphs were selected among the gene sets that were significantly correlated with altered genes in MK2i-NP-treated cells.

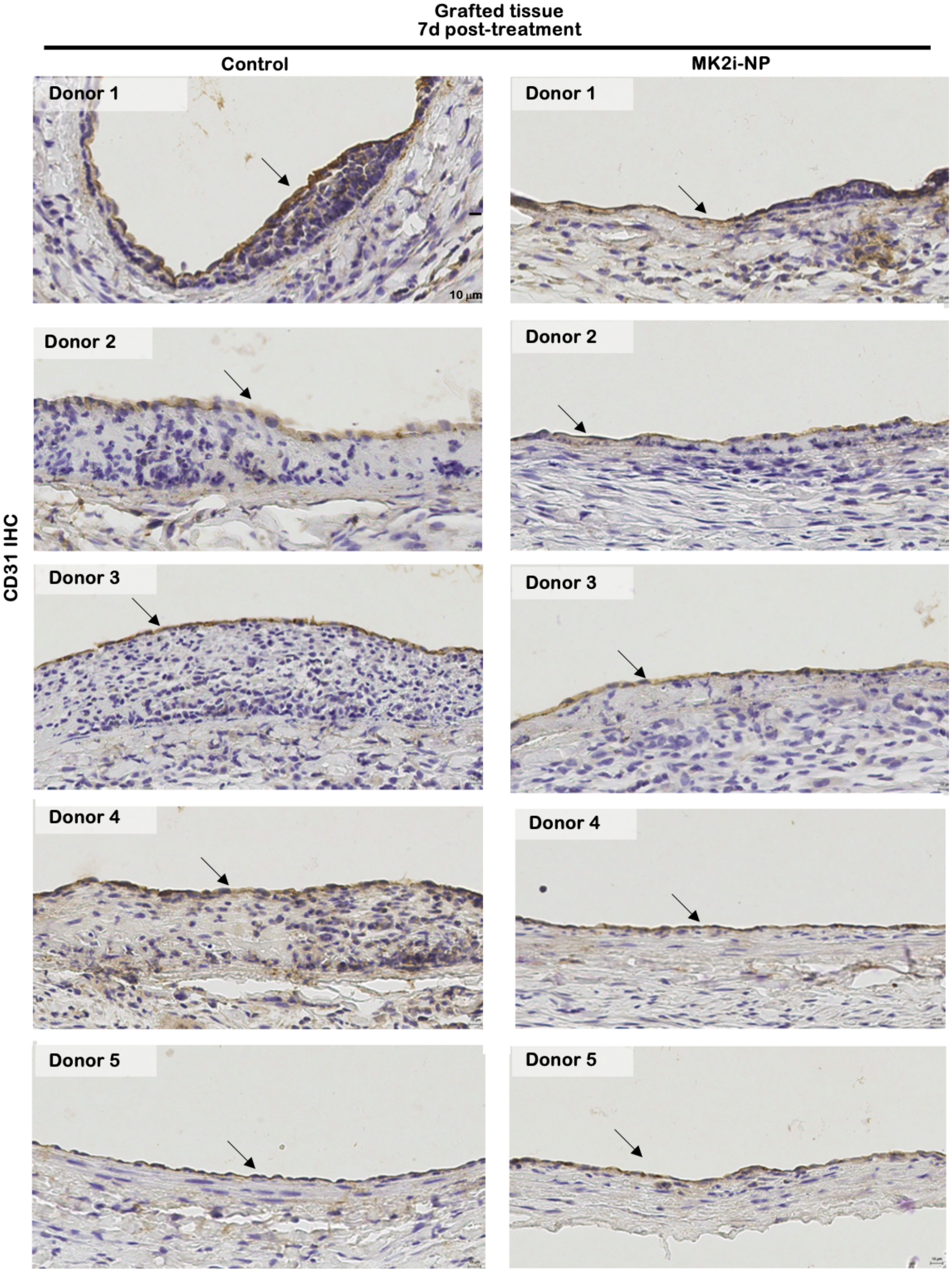

Figure S5. Rabbit vein grafts retain endothelial coverage after treatment and surgery. Rabbit external jugular veins were surgically excised, divided in half, treated 30 minutes with or without MK2i-NP$^{50:5Lyo}$, then surgically grafted onto contralateral carotid arteries of the same rabbit. After 7 days, grafted tissue was recovered and stained for CD31. Representative images from each grafted vein are shown.

Table S1. Statistical summary of ANOVA test done for 0.1% serum PDGF proliferation test.

Tukey's multiple comparisons test	Mean Diff.	95.00% CI of diff.	Significant?	Summary	Adjusted P Value
NT vs. NT + PDGF	-3.68	-5.317 to -2.044	Yes	****	<0.0001
NT vs. 100 µM MK2i	-3.36	-4.997 to -1.724	Yes	****	<0.0001
NT vs. 50 µM MK2i-NP	0.5057	-1.131 to 2.142	No	ns	0.8961
NT vs. 100 µM MK2i-NP	0.8109	-0.5925 to 2.214	No	ns	0.4632
NT + PDGF vs. 100 µM MK2i	0.32	-1.625 to 2.265	No	ns	0.9889
NT + PDGF vs. 50 µM MK2i-NP	4.186	2.241 to 6.131	Yes	****	<0.0001
NT + PDGF vs. 100 µM MK2i-NP	4.491	2.738 to 6.244	Yes	****	<0.0001
100 µM MK2i vs. 50 µM MK2i-NP	3.866	1.921 to 5.811	Yes	****	<0.0001
100 µM MK2i vs. 100 µM MK2i-NP	4.171	2.418 to 5.924	Yes	****	<0.0001
50 µM MK2i-NP vs. 100 µM MK2i-NP	0.3053	-1.448 to 2.058	No	ns	0.9862

Table S2. Statistical summary of ANOVA test done for A7r5 VSMC viability experiment.

Tukey's multiple comparisons test	Mean Diff.	95.00% CI of diff.	Significant?	Summary	Adjusted P Value
NT vs. MK2i	0.01946	-0.04338 to 0.08230	No	ns	0.8415
NT vs. MK2i-NPs	0.05197	-0.01087 to 0.1148	No	ns	0.1369
NT vs. Pos. Cont.	0.9735	0.9106 to 1.036	Yes	****	<0.0001
MK2i vs. MK2i-NPs	0.03252	-0.03032 to 0.09536	No	ns	0.5174
MK2i vs. Pos. Cont.	0.954	0.8912 to 1.017	Yes	****	<0.0001
MK2i-NPs vs. Pos. Cont.	0.9215	0.8587 to 0.9844	Yes	****	<0.0001

Table S3. Full one-way ANOVA results for CREB phosphorylation western blots on days 1, 3, 7, and 10.

**Day 1**					
Tukey's multiple comparisons test	Mean Diff.	95.00% CI of diff.	Significant?	Summary	Adjusted P Value
NT vs. NT + LPA	-1.556	-2.011 to -1.102	Yes	****	<0.0001
NT vs. MK2i + LPA	-0.9392	-1.394 to -0.4849	Yes	***	0.0008
NT vs. MK2i-NPs + LPA	-0.283	-0.7373 to 0.1713	No	ns	0.2659
NT + LPA vs. MK2i + LPA	0.6171	0.1627 to 1.071	Yes	*	0.0105
NT + LPA vs. MK2i-NPs + LPA	1.273	0.8190 to 1.728	Yes	****	<0.0001
MK2i + LPA vs. MK2i-NPs + LPA	0.6563	0.2020 to 1.111	Yes	**	0.0074
**Day 3**					
Tukey's multiple comparisons test	Mean Diff.	95.00% CI of diff.	Significant?	Summary	Adjusted P Value
NT vs. NT + LPA	-1.145	-1.449 to -0.8409	Yes	****	<0.0001
NT vs. MK2i + LPA	-0.8362	-1.140 to -0.5322	Yes	***	0.0001
NT vs. MK2i-NPs + LPA	-0.2537	-0.5578 to 0.05028	No	ns	0.1056
NT + LPA vs. MK2i + LPA	0.3087	.004716 to 0.6128	Yes	*	0.0466
NT + LPA vs. MK2i-NPs + LPA	0.8912	0.5872 to 1.195	Yes	****	<0.0001
MK2i + LPA vs. MK2i-NPs + LPA	0.5825	0.2784 to 0.8865	Yes	**	0.0013
**Day 7**					
Tukey's multiple comparisons test	Mean Diff.	95.00% CI of diff.	Significant?	Summary	Adjusted P Value
NT vs. NT + LPA	-1.391	-2.008 to -0.7733	Yes	***	0.0004
NT vs. MK2i + LPA	-1.348	-1.965 to -0.7305	Yes	***	0.0005
NT vs. MK2i-NPs + LPA	-0.6058	-1.223 to 0.01165	No	ns	0.0544
NT + LPA vs. MK2i + LPA	0.04281	-0.5746 to 0.6602	No	ns	0.9958
NT + LPA vs. MK2i-NPs + LPA	0.785	0.1676 to 1.402	Yes	*	0.0151
MK2i + LPA vs. MK2i-NPs + LPA	0.7422	0.1247 to 1.360	Yes	*	0.0204
**Day 10**					
Tukey's multiple comparisons test	Mean Diff.	95.00% CI of diff.	Significant?	Summary	Adjusted P Value
NT vs. NT + LPA	-1.444	-2.043 to -0.8456	Yes	***	0.0003
NT vs. MK2i + LPA	-1.468	-2.067 to -0.8698	Yes	***	0.0002
NT vs. MK2i-NPs + LPA	-0.8955	-1.494 to -0.2970	Yes	**	0.006
NT + LPA vs. MK2i + LPA	-0.02425	-0.6228 to 0.5743	No	ns	0.9992
NT + LPA vs. MK2i-NPs + LPA	0.5486	-0.04994 to 1.147	No	ns	0.0729
MK2i + LPA vs. MK2i-NPs + LPA	0.5728	-0.02569 to 1.171	No	ns	0.0607

Table S4. List of genes in KEGG_TGF_BETA_SIGNALING_PATHWAY, which was used to query RNA-Seq data of human primary coronary artery smooth muscle cells harvested from clinical samples treated with or without MK2i-NP were (N = 3 per group). An enrichment score for each curated gene for this KEGG Pathway was calculated using GSEA 4.1.0 software. The associated fold change for each gene is shown, as determined by RNA-Seq analysis, comparing expression changes in cells treated with MK2i-NP versus untreated cells.

GENE SYMBOL	Enrichment Score	Log2 Fold Change (MK2i-NP versus Control)	P-Value
THBS2	0.0352	-1.4651917	8.01E-08
BMP6	0.0699	-1.569711	1.93E-06
CDKN2B	0.0972	-1.1399918	0.00282506
LTBP1	0.1263	-1.0227961	8.50E-05
RBL2	0.1558	-0.8357188	0.00242486
SMAD3	0.1843	-0.8597901	0.00168497
INHBE	0.2101	-1.0714435	0.07986732
MYC	0.238	-0.7914765	0.00214905
SP1	0.2574	-0.6287946	0.00181864
TGFB3	0.2797	-2.4841598	5.71E-07
SMAD1	0.3007	-1.0901315	0.00191102
BMPR2	0.3223	-0.572002	0.01008074
TGFB2	0.3297	-1.5769365	0.00020903
TGFBR1	0.3463	-0.6129673	0.03114465
CHRD	0.3506	-0.9996664	0.04378031
COMP	0.3659	n.d.	n.d.
SMAD4	0.3731	-0.4125166	0.05024536
BMP8A	0.3903	n.s.	n.s.
THBS3	0.3959	n.s.	n.s.
BMPR1B	0.4109	n.s.	n.s.
SMAD5	0.4166	n.s.	n.s.
DCN	0.4031	n.s.	n.s.
ID4	0.415	n.s.	n.s.
CREBBP	0.4283	n.s.	n.s.
ROCK1	0.4324	n.s.	n.s.
EP300	0.4448	n.s.	n.s.
ZFYVE9	0.4546	n.s.	n.s.
ROCK2	0.464	n.s.	n.s.
RPS6KB1	0.4752	n.s.	n.s.
ZFYVE16	0.4806	n.s.	n.s.
ACVR2A	0.4812	n.s.	n.s.
BMPR1A	0.4509	n.s.	n.s.
E2F5	0.4067	n.s.	n.s.
BMP4	0.3275	n.s.	n.s.
GDF6	0.3219	n.s.	n.s.
ID3	0.3074	n.s.	n.s.
TGFBR2	0.3082	n.s.	n.s.
SMAD9	0.2955	n.s.	n.s.
ID2	0.2877	n.s.	n.s.
ACVR1C	0.2805	n.s.	n.s.

GENE SYMBOL	Enrichment Score	Log2 Fold Change (MK2i-NP versus Control)	P-Value
ACVR2B	0.2818	n.s.	n.s.
GDF7	0.2828	n.s.	n.s.
TGFB1	0.2696	n.s.	n.s.
ACVR1	0.2654	n.s.	n.s.
NODAL	0.2588	n.s.	n.s.
RHOA	0.26	n.s.	n.s.
SMAD2	0.2535	n.s.	n.s.
PITX2	0.2461	n.s.	n.s.
TFDP1	0.2464	n.s.	n.s.
SMAD6	0.231	n.s.	n.s.
RBL1	0.1752	n.s.	n.s.
SMURF1	0.1526	n.s.	n.s.
FST	0.1484	n.s.	n.s.
RBX1	0.1342	n.s.	n.s.
CUL1	0.1196	n.s.	n.s.
THBS4	0.1124	n.s.	n.s.
MAPK1	0.1036	n.s.	n.s.
GDF5	0.101	n.s.	n.s.
SMAD7	0.1001	n.s.	n.s.
ACVRL1	0.0935	n.s.	n.s.
PPP2R1B	0.0934	n.s.	n.s.
SMURF2	0.0679	n.s.	n.s.
E2F4	-0.0024	n.s.	n.s.
RPS6KB2	-0.0023	n.s.	n.s.
PPP2R1A	-0.0076	n.s.	n.s.
PPP2CA	-0.0147	n.s.	n.s.
INHBB	-0.0215	n.s.	n.s.
PPP2CB	-0.0214	n.s.	n.s.
ID1	-0.0303	n.s.	n.s.
THBS1	-0.0183	n.s.	n.s.
INHBC	-0.0123	n.s.	n.s.
INHBA	-0.0229	n.s.	n.s.
SKP1	-0.024	n.s.	n.s.
MAPK3	-0.0205	0.37510553	0.0710939
TNF	-0.0168	n.s.	n.s.
NOG	-0.0247	n.s.	n.s.
BMP8B	-0.0239	n.s.	n.s.
AMH	-0.0115	n.s.	n.s.
BMP2	0.0173	1.05002194	0.0009175

Table S5. List of genes in KEGG_ECM_RECEPTOR_INTERACTION, which was used to query RNA-Seq data of human primary coronary artery smooth muscle cells harvested from clinical samples treated with or without MK2i-NP were (N = 3 per group). An enrichment score for each curated gene for this KEGG Pathway was calculated using GSEA 4.1.0 software. The associated fold change for each gene is shown, as determined by RNA-Seq analysis, comparing expression changes in cells treated with MK2i-NP versus untreated cells.

Gene Symbol	Enrichment Score	Log2 Fold Change (MK2i-NP versus Control)	P-Value
LAMA5	1.852	-2.083186249	1.44E-12
COL5A2	1.621	-1.688775328	6.26E-08
THBS2	1.597	-1.46519165	8.01E-08
COL1A1	1.334	-1.612901168	8.70E-07
COL4A1	1.293	-1.00138327	0.000388
COL5A1	1.276	-1.66205986	1.60E-06
HSPG2	1.265	-1.462198497	7.16E-05
LAMA2	1.247	-1.437068621	5.84E-05
COL1A2	1.203	-1.391982992	7.56E-06
COL4A2	1.145	-0.895038811	0.00148
COL5A3	1.139	-2.547953268	0.002021
LAMB2	1.051	-0.759354914	0.003482
LAMC1	1.042	n.s.	n.s.
LAMB1	1.041	-1.030755447	0.000752
COL3A1	0.975	-2.387621616	4.24E-06
ITGAV	0.973	-0.629867351	0.014756
FN1	0.944	-1.044944594	0.020816
ITGA4	0.914	-0.604567027	0.036033
COL4A4	0.91	-1.841605114	0.001085
ITGA1	0.908	-1.134842949	0.003289
COL4A6	0.897	-1.135307151	0.003179
COL6A3	0.88	-1.170899677	0.001265
ITGA8	0.855		
AGRN	0.85	-0.847987373	0.007853
COL6A1	0.831	-0.805428447	0.009089
COL6A2	0.831	-0.834331833	0.008532
VWF	0.82	-2.964830426	0.000679
LAMA4	0.779	-0.740264755	0.017725
ITGA10	0.779	-0.789340112	0.045694
COMP	0.746	n.d.	n.d.
VTN	0.736	n.s.	n.s.
ITGB8	0.727	-1.772523292	0.001398
ITGA9	0.681	n.s.	n.s.
THBS3	0.664	n.s.	n.s.
COL6A6	0.648	n.s.	n.s.
ITGA6	0.588	n.s.	n.s.
ITGB1	0.547	n.s.	n.s.
DAG1	0.442	n.s.	n.s.
ITGA2B	0.387	n.s.	n.s.

Gene Symbol	Enrichment Score	Log2 Fold Change (MK2i-NP versus Control)	P-Value
ITGA11	0.376	n.s.	n.s.
HMMR	0.373	n.s.	n.s.
TNXB	0.362	n.s.	n.s.
SV2A	0.332	n.s.	n.s.
CD47	0.318	n.s.	n.s.
SDC3	0.282	n.s.	n.s.
TNC	0.264	n.s.	n.s.
LAMA3	0.241	n.s.	n.s.
SDC2	0.234	n.s.	n.s.
TNR	0.201	n.s.	n.s.
LAMC2	0.088	n.s.	n.s.
ITGB5	0.001	n.s.	n.s.
COL11A2	-0.033	n.s.	n.s.
ITGA5	-0.069	n.s.	n.s.
LAMB3	-0.095	n.s.	n.s.
GP6	-0.12	n.s.	n.s.
THBS4	-0.13	n.s.	n.s.
ITGB6	-0.166	n.s.	n.s.
COL2A1	-0.198	n.s.	n.s.
ITGA7	-0.2	n.s.	n.s.
ITGA3	-0.213	n.s.	n.s.
GP5	-0.219	n.s.	n.s.
CHAD	-0.243	n.s.	n.s.
CD36	-0.267	n.s.	n.s.
LAMC3	-0.27	n.s.	n.s.
RELN	-0.299	n.s.	n.s.
LAMA1	-0.308	n.s.	n.s.
COL11A1	-0.313	n.s.	n.s.
ITGB4	-0.332	n.s.	n.s.
LAMB4	-0.398	n.s.	n.s.
ITGA2	-0.419	n.s.	n.s.
THBS1	-0.493	n.s.	n.s.
GP1BA	-0.609	n.s.	n.s.
SDC4	-0.763	n.s.	n.s.
ITGB3	-0.786	0.954969832	0.006175
ITGB7	-1.128	n.s.	n.s.
SDC1	-1.205	0.704474092	0.003958
SPP1	-1.216	4.902028593	3.93E-05
CD44	-1.344	0.790959797	4.59E-05
